# On the Aperture Problem of Binocular 3D Motion Perception

**DOI:** 10.3390/vision3040064

**Published:** 2019-11-19

**Authors:** Martin Lages, Suzanne Heron

**Affiliations:** 1 School of Psychology, University of Glasgow, Glasgow G12 8QB, UK; zancan7@hotmail.com; 2 Psychological Institute, University of Tübingen, D-72076 Tübingen, Germany

**Keywords:** velocity, disparity, local motion, Bayesian inference

## Abstract

Like many predators, humans have forward-facing eyes that are set a short distance apart so that an extensive region of the visual field is seen from two different points of view. The human visual system can establish a three-dimensional (3D) percept from the projection of images into the left and right eye. How the visual system integrates local motion and binocular depth in order to accomplish 3D motion perception is still under investigation. Here, we propose a geometric-statistical model that combines noisy velocity constraints with a spherical motion prior to solve the aperture problem in 3D. In two psychophysical experiments, it is shown that instantiations of this model can explain how human observers disambiguate 3D line motion direction behind a circular aperture. We discuss the implications of our results for the processing of motion and dynamic depth in the visual system.

**Dataset:** osf.io/2j6sq.

## 1. Introduction

Local lines and edges are elementary for retinal image encoding and processing because they indicate a change in reflectance of a surface, a change in the amount of light falling on it, or a change in surface orientation relative to the light source of an object. Static as well as moving line segments are encoded by specialised cells within local receptive fields [1]. These lines and contours are regarded as important image-based features, providing the primitives for object and scene recognition in visual systems [2]. However, it is impossible to recover the true 2D velocity from a line moving inside a circular aperture. This is because tangential motion along the line, without visible endpoints, remains ambiguous. Computing the velocity perpendicular to a moving line or edge provides a default solution to the 2D aperture problem but may not coincide with the true stimulus motion [3,4]. In the following a velocity measured on the retina or screen, that is perpendicular to a moving line, is called image-motion gradient.

In the absence of additional features human observers typically perceive 2D motion of a line stimulus inside a circular aperture in the direction of the shortest path or image-motion gradient. This well-established finding on 2D motion perception [5,6], together with results on the 2D aperture problem [7], suggests that minimal displacement is a general principle when establishing 2D motion percepts [8,9]. To the best of our knowledge this has not been established for 3D motion perception.

In many studies researchers have addressed the aperture problem of 2D motion perception [7,10,11,12,13] but surprisingly few have investigated the equally pertinent aperture problem of 3D motion perception [14]. In these studies researchers have employed lines or plaids moving at a constant depth behind an aperture [15,16] or random-dots on a surface moving in depth along the line of sight [17,18]. Here we investigate the perceived 3D motion direction of a single line stimulus moving behind a circular aperture on a horizontal or oblique trajectory in depth. We will make three contributions towards the understanding of 3D motion perception: First, we illustrate the ambiguity of local line motion direction in the context of two geometric default strategies. Second, we suggest a probabilistic approach to the 3D aperture problem by combining binocular velocity constraints with an ecological-plausible 3D motion prior. Third, we investigate perceived 3D trajectories of line stimuli behind an aperture by systematically varying the 3D orientation of the line.

### 1.1. The Inverse Problem and Geometric Defaults

In the following we assume that an observer maintains fixation on a point straight ahead at the centre of a circular aperture. A detailed mathematical treatment of the binocular viewing geometry, resulting velocity constraints, and probabilistic models are provided in Appendixes Appendix A.1–Appendix A.4. Throughout the text we will refer to relevant equations in the appendices (Equations (A1)–(A19)).

If a single line stimulus moves on a trajectory in 3D and is viewed through an aperture without visible endpoints then its trajectory remains ambiguous. The orientation of a moving line stimulus and its projection into the left and right eye determines local 2D motion encoding on the back of each eye- and equivalently the stereoscopic images on an image plane in front of the observer (Equations (A3) and (A4)). If the line stimulus is vertical and moves on the horizontal plane in depth then the image-motion gradients in the left and right eye may have different speeds and therefore interocular velocity difference (IOVD) but share the same horizontal (epipolar) motion direction. As a consequence of this horizontal alignment, inverse projection of the image gradients through the nodal point of the left and right eye intersect in a single point in 3D. Together with the start point of stimulus motion, geometric triangulation in depth (ray-tracing) results in a uniquely defined 3D motion vector. If, however, the moving line stimulus is not vertical but tilted and/or slanted in 3D then inverse projections of the image-motion gradients do not necessarily intersect. Simple ray-tracing breaks down and alternative strategies are required to disambiguate the 3D aperture problem [19].

**Figure 1:**
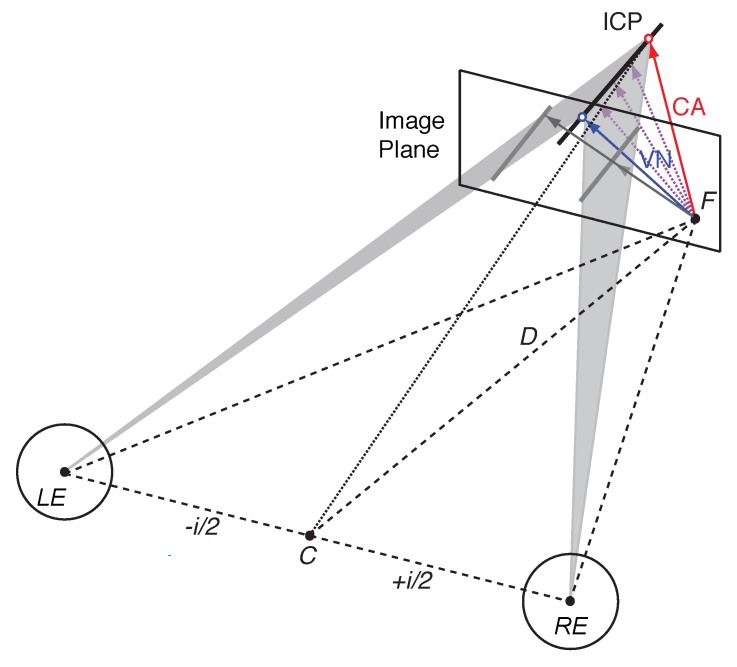
Illustration of velocity constraints and the 3D aperture problem. The left eye (LE) and right eye (RE), with nodal points separated by interpupillary distance *i* (exaggerated here), fixate point *F* on an image plane (screen) at distance *D*. Stereoscopic rendering of a tilted moving line on the image plane (grey lines) suggests two image-motion gradients (grey arrows) originating from point *F*. Inverse projection of each image gradient through the nodal points of each eye do not intersect but the intersection of velocity constraint planes (shaded triangles) describe all possible motion directions in a single line (ICP). Different 3D velocity estimates are shown in colour (blue—VN: Vector Normal; red—CA: Cyclopean Average; light purple – intermediate directions, see text for explanation).

The binocular viewing geometry for an oriented line stimulus moving in depth is illustrated in Figure 1. Local encoding gives rise to 2D velocity constraint lines on the back of the left and right eye, or equivalently constraint lines on a fronto-parallel image plane at viewing distance *D*. Inverse projection of these 2D constraint lines through the nodal point of the left (LE) and right eye (RE) describe two constraint planes cutting through 3D space (shaded triangles in Figure 1; Equation (A8)). If a moving line stimulus is projected into the left and the right eye then each of these velocity constraint planes describe all possible line positions, orientations and velocities in 3D. Each constraint plane is the result of monocular 2D line motion detection and inverse projection of retinal motion through the corresponding nodal point. If the two constraint planes are not parallel then they intersect in a single line in 3D [20]. The intersection of the left and right constraint plane, or ICP for short, coincides with a new position of the moving line stimulus in 3D space after a short time interval. Thus, the ICP describes all possible 3D velocities that satisfy both the velocity constraint in the left as well as the right eye, effectively describing a binocular velocity constraint in 3D (Equation (A7)). However, similar to the 2D aperture problem, motion along the ICP itself remains ambiguous. Since an infinite number of vectors connect the start point with the ICP, the visual system requires additional information to establish a unique motion percept.

### 1.2. Vector Normal and Cyclopean Average

If a line stimulus translates from a start point to a new position then a single 3D vector describes its veridical or true 3D velocity. The vector normal (VN) is the shortest vector in 3D that connects the start point with the line at its new position. By definition the VN is orthogonal to the orientation of the line stimulus and describes the shortest motion path (blue arrow in Figure 1). If the binocular viewing geometry is known then the VN can be computed from the velocity constraints in the left and right eye (Equation (A6)).

An alternative geometric strategy is to compute the 2D image gradients in the left and right eye and to fuse them by averaging the two vectors. Inverse projection of this average gradient through the cyclopean point *C*, the midpoint between the left and right eye, results in a cyclopean constraint line for 3D motion direction (dotted line in Figure 1). This can be achieved, for example, by projecting the image gradient of the left and right eye on a common image plane and by taking the vector average of both vectors in that image plane. The average image gradient gives rise to a robust constraint because it is based on independent motion detection in each eye. Assuming that the 3D motion system can establish the intersection between ICP and the cyclopean constraint line, the cyclopean average (CA) also provides a unique 3D velocity estimate (red arrow in Figure 1). This estimate reflects the constant IOVD between the image gradients (see [19] for details).

The VN and CA strategy are geometric models and therefore parameter-free. They are inspired by the intersection of constraints (IOC) and vector average (VA) strategy that have been applied to the 2D aperture problem. Since VN and CA both use motion representations in a binocular viewing geometry, it is not possible to establish these geometric defaults from monocular motion input only. Both strategies require knowledge of the binocular viewing geometry. Furthermore, selecting either VN or CA seems insufficient as both geometric defaults may not coincide with the motion direction perceived by an observer nor the true (veridical) motion direction of the stimulus. Establishing more flexible models is critical because the true motion direction of a stimulus may only be revealed after local and global motion and disparity constraints have been integrated.

### 1.3. Bayesian Inference

Perception may be understood as an inference because it solves the inverse problem of perception by estimating distal stimulus characteristics from proximal cues [21,22]. More specifically, Bayesian inference combines uncertain sensory information extracted from images on the retina with prior assumptions about the nature of objects in the world [23,24,25]. This approach has been successful when modelling human visual perception and performance in a range of psychophysical tasks [26].

In a seminal paper on motion perception Weiss, Simoncelli and Adelson [27] put forward a Bayesian observer model that infers perceived direction and speed in 2D images. This probabilistic model assumes that local motion filters establish velocity constraints with different degrees of uncertainty. Combining these constraints with a weak motion prior suggests a maximum a posteriori motion estimate. However, Bayesian 2D models [27,28,29] are limited to 2D motion on a plane. Related models on motion in depth [30,31,32] studied motion on a *x*-*z* plane assuming pixel- or point-wise encoding and corresponding (retinal) 2D motion priors. As a result these models are limited to planar motion of points and cannot explain arbitrary 3D motion trajectories of lines.

**Figure 2:**
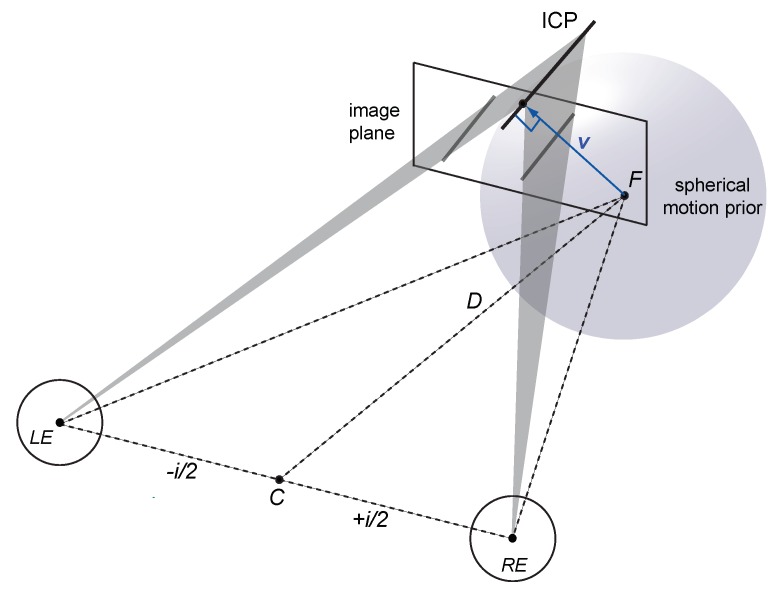
Illustration of the Bayesian vector normal (BVN) model. Inverse projection of the line stimulus into the left and right eye are illustrated by 2D velocity constraints on an image plane (grey lines), resulting in two 3D velocity constraint planes (shaded triangles), and their intersection (ICP). The ICP describes an oriented line moving from the start point *F* to the left and away from the observer. The translucent sphere centred on fixation point *F* denotes a weak prior for slow motion in 3D. If the constraint planes have very little noise and are combined with the spherical prior in a Bayesian inference then the resulting 3D velocity estimate $\hat{v}$ approximates the vector normal (blue arrow).

Here we address the 3D aperture problem [14,15] by extending probabilistic 2D models of binocular motion perception to arbitrary 3D trajectories of lines. The proposed model of 3D motion perception assumes that (i) 3D velocity constraints arise from inverse projections of local image velocity constraints in a binocular viewing geometry, (ii) noise from monocular motion and dynamic binocular disparity processing is independent and additive, and (iii) motion perception prefers slow motion trajectories in 3D (see Figure 2 and Appendix A.4). This leads to a number of interesting observations.

Similar to the geometric VN strategy for 3D line motion, the Bayesian vector normal (BVN) relies on inverse projections of velocity constraints in the left and right eye. The intersection of the left and right eye velocity constraint planes results in a binocular constraint line in 3D. Importantly, this intersection of constraint planes (ICP) can be derived from inverse projections of monocular motion constraints as well as binocular disparity constraints monitored over time [33,34]. In the following we assume that both inputs, monocular motion and binocular disparity over time [35], are integrated by a 3D motion system but have independent sources of noise (Equations (A10) and (A11)). This is a plausible assumption because it is well known that the visual system receives input from monocular motion [1,36] as well as binocular disparity processing [37,38].

### 1.4. Spherical Motion Prior

Weiss, Simoncelli and Adelson [27] proposed a bivariate (2D) Gaussian motion prior in the image plane to explain bias in 2D motion perception. Here we use minimal distance as a general principle and apply it to the binocular velocity constraints in 3D. If we disregard self-motion and eye movements then most features and objects in a scene remain stationary or move relatively slowly [8,9]. As a consequence, a spherical Gaussian centred on the start point of a moving stimulus provides an ecologically plausible prior for 3D motion in the world (Equation (A13)). Please note that a spherical motion prior is conceptually different from a 2D motion prior because a world prior should scale with viewing distance. In contrast, a 2D retinal motion prior [25,27,28,29] would affect motion close to an observer in the same way as equivalent motion viewed from a distance and vice versa. In other words, if the motion system does not take into account viewing distance between the moving object and the observer then Bayesian estimation would lead to implausible 3D motion predictions. A model with a 3D motion prior on the other hand makes ecologically plausible 3D velocity predictions [33,34,39].

### 1.5. Bayesian Vector Normal

The Bayesian vector normal (BVN) model combines noisy velocity constraints from the left and right eye with a spherical motion prior (Equation (A14)) at a given viewing distance to resolve the 3D aperture problem. If the velocity constraints of the Bayesian vector normal model have the same noise parameter σ in all three spatial components *x*, *y* and *z* (BVN1) then the ratio between noise σ of the velocity constraints and noise $\sigma_{0}$ of the spherical prior characterises the perceived motion direction of an oriented line stimulus behind an aperture. A weak motion prior with a small noise ratio between noise and prior (σ:$\sigma_{0}\;<\;0.01$) minimises displacements of the line stimulus in 3D, thereby approximating the vector normal (VN) strategy. The BVN1 model with the same noise parameter in all three spatial components indicates a single noise source as implied by computational models with motion input only [31,40,41] and models with joint encoding of motion and disparity [42,43,44,45].

A Bayesian vector normal model with two independent noise parameters (BVN2) distinguishes between noise $\sigma_{m}$ in the *x*-*y* image plane and noise $\sigma_{d}$ along the *z*-component. The two noise parameters in the BVN2 model suggest two separate processing streams with independent sources of noise [35]: (1) Noise along the image plane *x*-*y* relates to uncertainty in 2D motion processing, and (2) noise along the *z*-axis, at the intersection of the monocular velocity constraint planes, reflects uncertainty in depth processing over time. If we assume that the prior is constant then the two noise parameters quantify how strongly perceived motion directions are the result of uncertainty in monocular motion and uncertainty in dynamic depth processing. More specifically, in the context of line motion inside an aperture, noise in the *x*-*y* plane may reflect ambiguity in monocular 2D motion processing whereas noise in dynamic depth along the *z*-axis may reflect ambiguity in dynamic binocular disparity processing.

Both the BVN1 and BVN2 model explain perceived 3D motion direction by combining likelihood constraints with a weak isotropic motion prior and finding the maximum a posterior velocity estimate (Equation (A16)). The predictions of the BVN1 model with a single noise parameter deviate from the predictions of the vector normal (VN) strategy with increasing noise levels. The BVN2 model with two parameters is more flexible than BVN1 and can approximate the vector normal (VN), the cyclopean average (CA), as well as intermediate 3D motion directions. By fitting the BVN2 to observed motion directions we can quantify the noise ratios between likelihood and prior for each individual observer. If the noise ratios for motion and dynamic depth processing are significantly different from each other then it seems likely that two independent noise sources contribute to the perception of 3D motion.

More specifically, if the noise for monocular motion processing is smaller than the noise for dynamic depth processing $\sigma_{m}<\sigma_{d}$ then 3D motion perception receives stronger input from monocular motion processing than dynamic depth processing [31]. Alternatively, if the noise for monocular motion processing is larger than the noise for dynamic depth processing $\sigma_{m}>\sigma_{d}$ then dynamic depth processing contributes more strongly than monocular motion processing. Two independent noise sources imply separate motion and dynamic depth processing streams, as has been suggested for motion and dynamic disparity processing [30,46,47,48].

In two experiments we manipulated 3D orientation (slant and tilt) of a single line stimulus moving behind a circular aperture. This stimulus manipulation can reveal whether 3D motion perception is better captured by the deterministic VN and CA strategy or by more flexible probabilistic models such as BVN1 and BVN2. For oriented line stimuli with a fixed (non-zero) interocular velocity difference (IOVD) as in the present experiments the CA strategy predicts a single 3D motion direction for different line orientations whereas the VN strategy predicts diverging 3D motion directions.

## 2. General Methods

### 2.1. Participants

All participants had normal or corrected-to-normal visual acuity and were screened for stereo deficiencies (Random Dot E test). We recruited a total of six participants. Four participants were students from Glasgow University (S2, S4, S5, and S6) who were naive about the aims of the experiments and two were the authors (S1 and S3). Participant S6 showed no deficits in the stereo screening but was excluded from testing because depth discrimination was close to chance, even after several training blocks. Before testing commenced each observer attended at least two training blocks and received acoustic feedback when adjustment of the depth probe was opposite in depth (near/far) to the actual motion direction in depth. The feedback established an exclusion criterion (not significantly above 50% correct) and helped observers to improve their performance. Informed written consent was obtained from all naive observers before participation. The experimental procedures were approved by the College for Science and Engineering Ethics Committee at Glasgow University and were in compliance with national legislation and the Code of Ethical Principles for Medical Research Involving Human Subjects of the World Medical Association (Declaration of Helsinki).

### 2.2. Apparatus

The stimulus and task were programmed in MATLAB (MathWorks, Natick, MA, USA) using the Psychophysics Toolbox extensions [49,50]. The experiments were run on a Macintosh G4 computer with two 21 in Sony GDM-F500R cathode-ray tube flat-screen monitors viewed through two mirrors in a Wheatstone configuration. The monitors were calibrated using a Minolta photometer (Cambridge Research Systems, Rochester, UK). Stimuli were presented in stereoscopic view at a viewing distance of 55 cm on each monitor screen. Stimuli were shown with 50% Michelson contrast and at a refresh rate of 120 Hz. Observers were comfortably seated on height-adjustable chairs in front of the two mirrors with their head supported by a chin- and head-rest (UHCO Houston, TX, USA). The experimental room remained dark with lights switched off during testing.

### 2.3. Stimulus

**Figure 3:**
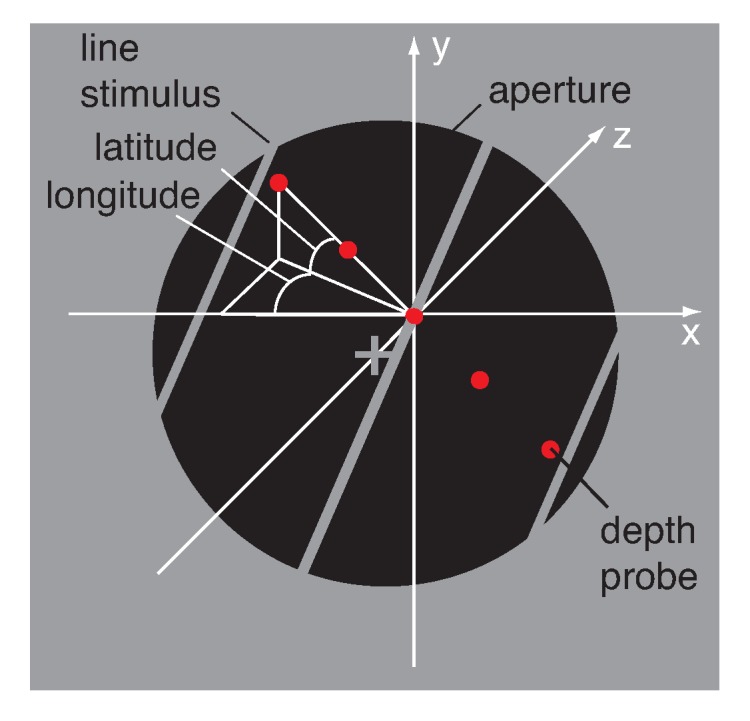
Illustration of the stimulus display and adjustment method. White hairlines indicate the axes (*x*, *y*, and *z*) and angles (longitude, latitude) in 3D and were not visible in the display. In each trial an oriented line stimulus moved back and forth on a 3D trajectory behind a circular aperture. The three grey lines illustrate a line stimulus at its far (left), start (centre), and near (right) position. The observer adjusted a string of five red dots until they matched the perceived 3D motion direction of the moving line stimulus. Motion direction was expressed in longitude and latitude angle, measured in degrees from the midpoint of the depth probe. See text for details.

As illustrated in Figure 3 observers fixated a hairline cross at the centre of a black circular aperture with a radius of 2.41 deg surrounded by a uniform mid-grey display of 25 cd/m${}^{2}$. Vertical nonius lines above and below the fixation-cross helped to maintain vergence. An oriented line of the same mid-grey as the surround moved inside the circular aperture. The line images and circular aperture on the left and right screen were anti-aliased (Gaussian half-width of 4 pixels) and blended perfectly with the grey surround, revealing no endpoints of the line stimulus. The line images on the left and right screen had different orientations giving rise to the perception of a 3D line stimulus slanted about the horizontal axis. Perceived slant can be described as optical (relative to the line of sight), geographical (relative to the gravitational vertical), or relative to another surface. In the present experiments optical slant coincided with geographical slant. Slant was therefore defined as the angular degree at which a plane was rotated from the fronto-parallel image plane, whereas tilt was defined as the axis of rotation for slant.

Due to the sign of the IOVD between the line images in the left and right eye the binocularly fused 3D line stimulus moved between inflection positions on the near left and far right (+IOVD) or the near right and far left (−IOVD) behind the circular aperture. All perceived motion directions were reported in spherical angles of longitude and latitude measured from the start point of the line stimulus behind the aperture (Equations (A1) and (A2)). Line stimuli with positive interocular velocity difference (+IOVD/near) were assigned positive longitude angles whereas line stimuli with negative interocular velocity difference (−IOVD/far) were assigned negative longitude angles. Longitude angle α was measured in degrees (deg) from the horizontal *x*-axis. Latitude angle β was measured in degrees from the horizontal *x*-*z* plane at a longitudinal angle α. Latitude angles were positive for trajectories pointing upwards relative to the line of sight and negative for trajectories pointing downwards.

The line images on the left and right CRT monitor moved with an average velocity of 3.0 deg/s measured on the fronto-parallel image plane along the horizontal axis in Exp. 1 and oblique axes in Exp. 2. All line stimuli had a horizontal (Exp. 1) or oblique (Exp. 2) velocity difference of ±0.46 deg/s on the image plane. Although line stimuli were perceived to travel on different 3D trajectories, their IOVD remained constant.

### 2.4. Procedure

**Figure 4:**
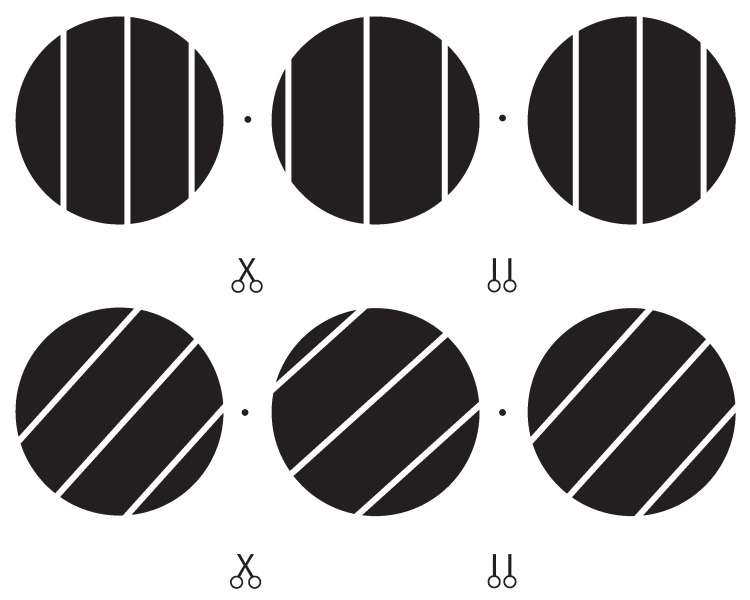
Stereoscopic illustration of the vertical line stimulus with horizontal disparity in the top row (Exp. 1) and an oblique line stimulus with horizontal and orientation disparity in the bottom row (Exp. 2). Cross-fusing the two dots to the left and right of the middle aperture or fusing the middle and right aperture (uncrossed fusion) gives an impression of the 3D orientation of the line stimulus at a “far”, “centre”, and ”near” position. In each trial the line stimulus moved back and forth between the near and far inflection position, suggesting a motion direction in 3D.

Perceived 3D motion direction of a single line stimulus was measured in a matching task without time limit. After observing the moving line stimulus behind the aperture the observer initiated the matching procedure by pressing the space bar on the keyboard. Five red dots, aligned and equally spaced in 3D, appeared inside the aperture and served as a probe to indicate 3D motion direction (see Figure 4). The observer could toggle between appearance and disappearance of the string of dots without disrupting the display of the moving line stimulus.

In each trial the observer carefully adjusted orientation and depth of the probe dots until they matched the perceived 3D motion direction of the slanted line stimulus. Successful adjustment of the probe was achieved when the moving line stimulus visited each probe dot without noticeable spatial offset and shearing in 3D. Once the observer was confident that the probe matched the perceived motion direction of the line stimulus they confirmed their settings and initiated the next trial. A typical trial lasted between 10 s and 15 s.

Although each probe dot should have unambiguous horizontal disparity, depth in the probe was not perceived veridically. We therefore assessed perceived depth in the probe in an additional block of twenty trials. Each observer first adjusted the probe dots to a perceived 3D motion direction while looking through the stereoscope. Then they indicated perceived longitude (at 0 deg latitude) of the probe dots using an external protractor device in normal binocular view. Observers consistently underestimated depth in the probe dots by an average factor of 0.6. This is in line with previous psychophysical studies that measured perceived slants of stereoscopically rendered static stimuli. Reduced depth of surfaces or stimuli has been observed in conditions with only a few relative depth cues [51,52] and possible cue conflicts in the stimulus display [53]. All horizontal disparity settings of the probe dots were scaled by this factor before transforming the data into longitude and latitude angle using a ray-tracing algorithm. In the results we report longitude and latitude angles after disparity scaling because the adjusted angles gave a more plausible account of perceived motion directions This scaling did affect absolute noise but crucially not the relative noise of BVN2 model estimates.

## 3. Results

**Figure 5:**
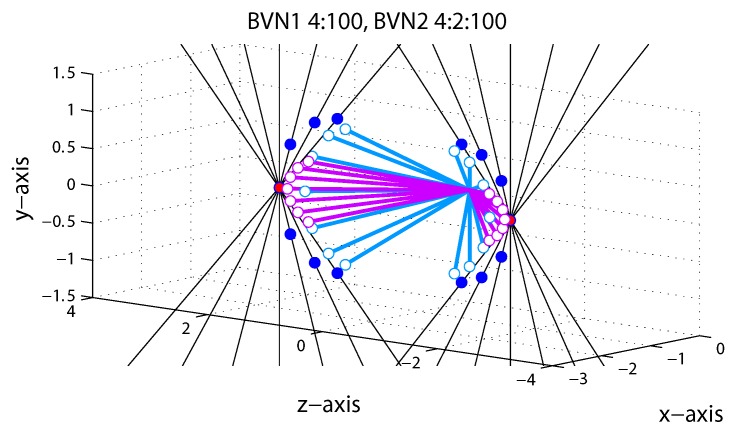
Model predictions for line stimuli in Exp. 1. Each of the seven black hairlines corresponds to a constraint (ICP) for a motion trajectory to the left and away from the observer (−IOVD/far) and to the left and towards the observer (+IOVD/near). Red dots correspond to the cyclopean average (CA) and dark blue dots to vector normal (VN) predictions. Light blue and light purple open circles correspond to BVN1 and BVN2 predictions, respectively. See text for details.

We focussed on perceived motion direction of the line stimuli in terms of perceived longitude and latitude angles. We did not measure perceived speed because speed of 3D motion stimuli is notoriously difficult to measure. Manipulation of orientation disparity in the line stimulus and the resulting model predictions are illustrated in Figure 5 for Exp. 1. The observer was positioned on the *z*-axis at −55 cm from fixation and viewed the oriented line stimulus moving on a trajectory in depth through a circular aperture. In each trial a single oriented line moved through the co-ordinates (0,0,0) to a near or far position on the left or right. Each of the oriented thin black lines in Figure 5 describe one of seven constraint lines (ICPs), shown for the far left position (−IOVD/far) and near left position (+IOVD/near) only.

The cyclopean average (CA) predicts constant horizontal motion trajectories (red dots in Figure 5) for all seven line stimuli whereas the vector normal (VN) predicts diverging motion trajectories on a curve (dark blue dots in Figure 5). The latter describes the shortest paths from the start point (0,0,0) to each constraint line [19]. For vertical lines with zero orientation disparity VN and CA predictions are identical. Predictions of BVN1 and BVN2 are shown as lines with open circles attached indicating intermediate 3D motion trajectories. The BVN1 predictions (light blue) with small noise relative to the prior (σ:$\sigma_{0}$ < 1:100) approximate the vector normal (VN) trajectories. For increasing noise levels BVN1 predicts shorter vectors with reduced longitude angles. In Figure 5 they are illustrated for a noise ratio of σ:$\sigma_{0}$ = 4:100. For BVN2 with noise ratios $\sigma_{m}$:$\sigma_{d}$:$\sigma_{0}$ = 4:2:100 (light purple) the motion noise is twice as large as the depth noise (4:2). The BVN2 predicts trajectories that diverge less and are more tightly packed around the CA than for BVN1. For a weak spherical motion prior (100) and small but increasingly unbalanced noise $\sigma_{m}$ > $\sigma_{d}$ BVN2 approximates constant CA predictions. Conversely, if depth noise exceeds motion noise $\sigma_{d}$ > $\sigma_{m}$ the BVN2 trajectories approximate the diverging 3D motion trajectories of the VN strategy. If the two noise parameters of the BVN2 model are sufficiently similar ($\sigma_{m}\approx \sigma_{d}$) then BVN2 makes equivalent predictions to the BVN1. Although BVN2 is more flexible than BVN1, both models are constrained by the minimal distance principle, resulting in a characteristic curve of diverging motion trajectories. (The reader can explore predictions for different noise settings using the MatLab code provided under osf.io/2j6sq).

### 3.1. Results of Experiment 1

In Exp. 1 line stimuli with orientation disparity were slanted in depth from vertical about the horizontal. Despite the ambiguity in the moving line stimuli all observers had similar characteristics in their perceived motion directions. Adjustments of all observers deviated systematically from the vector normal (VN) as well as the cyclopean average (CA) strategy. Large deviations between the data and predictions of the geometric default strategies made any goodness-of-fit and model comparison unnecessary. In the following we report best BVN1 and BVN2 model fits to the perceived motion directions for each participant. They are expressed in terms of longitude and latitude angles (Equations (A1) and (A2)) for 14 line stimulus conditions (2 IOVDs × 7 orientation disparities). We compared individual BVN1 and BVN2 fits using a conventional likelihood ratio test (LR) and an approximation of the Bayes factor (BF) (see Appendix A.5 for details).

**Figure 6:**
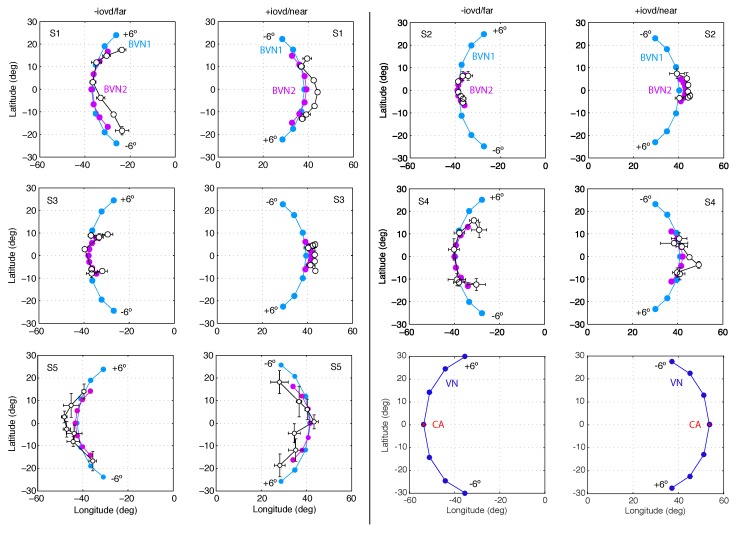
Motion directions in Experiment 1. In two columns pairs of graphs are shown for Observer S1 to S5 with −IOVD/far on the left +IOVD/near on the right. In each graph latitude is plotted against longitude angles. Circles connected by a line indicate motion directions for the seven line stimuli slanted in depth from vertical with orientation difference $-6^{{}^{\circ}}$ to $+6^{{}^{\circ}}$ in steps of $2^{{}^{\circ}}$. Black open circles represent observed longitude and latitude angles, averaged across repeated trials. Horizontal and vertical error bars denote ±1 SEM. Light blue and light purple circles denote motion direction estimates of the best-fitting BVN1 and BVN2 model, respectively. The last pair illustrates geometric predictions of VN (blue circles) and CA (red circles).

BVN2 parameter estimates for motion noise $\sigma_{m}$ were larger than for depth noise $\sigma_{d}$ and BVN2 explained the data of Observer S2 and S3 significantly better than BVN1 (see Figure 6 and Table A1). Expressing noise in the likelihoods as a percentage of the noise in the motion prior (100%), Observer S1 had 8.9% motion noise along the *x*-and *y*-axis and 6.8% depth noise along the *z*-axis. For the BVN1 model the noise estimate was 7%. Comparing the BVN models for Observer S1 the likelihood ratio test (LR) showed no statistically significant difference ($\chi^{2}\left(1\right)$ = 1.89, *p* = 0.17) and the Bayes factor (BF = 0.69) indicated negligible evidence in favour of BVN1. Observer S2, on the other hand, had 14.5% motion noise and 6.0% dynamic depth noise for BVN2 compared to 6.4% noise in the BVN1 model. The likelihood ratio between these model fits was statistically significant ($\chi^{2}\left(1\right)$ = 8.27, *p* = 0.004) and the Bayes factor (BF = 16.7) indicated strong evidence in favour of BVN2. A similar effect emerged for Observer S3 ($\chi^{2}\left(1\right)$ = 5.08, *p* = 0.024; BF = 3.4). Observer S4 ($\chi^{2}\left(1\right)$ = 3.28, *p* = 0.07; BF = 1.4) and Observer S5 ($\chi^{2}\left(1\right)$ = 3.37, *p* = 0.07; BF = 1.4) followed the same trend but their likelihood ratios were not statistically significant.

**Figure 7:**
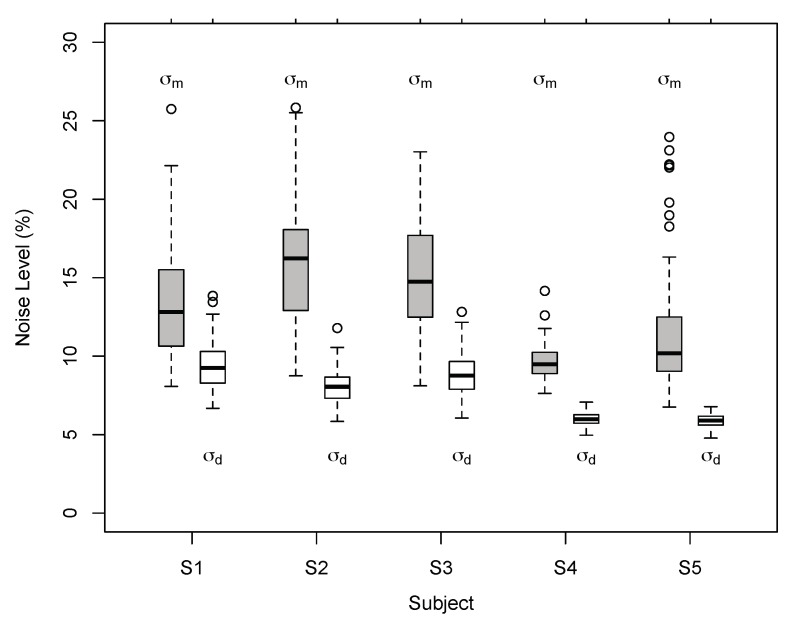
Individual noise estimates of BVN2 in Exp. 1. Monocular motion ($\sigma_{m}$) and dynamic depth ($\sigma_{d}$) estimates for Observer S1 to S5 from bootstrapped data. Thick horizontal lines denote medians, boxes the second to third quartile with error bars (whiskers) extending 1.5 times of the interquartile range. Open circles denote outliers. All pairwise differences between individual motion and depth noise estimates were statistically significant (*p* < 0.002, two-tailed *t*-test, Bonferroni-adjusted).

The results of Exp. 1 suggest that perceived 3D line motion directions deviated systematically from geometric default strategies such as the vector normal (VN) and cyclopean average (CA). Perceived motion directions varied across observers but had characteristics that are better described by a probabilistic model than geometric strategies. Model comparisons favoured the BVN2 over the BVN1 model in two observers. Two more observers showed a non-significant trend in favour of the BVN2 model whereas the result for one observer was inconclusive. Nevertheless, noise ratios $\sigma_{m}$:$\sigma_{0}$ and $\sigma_{d}$:$\sigma_{0}$ for the BVN2 model indicated that uncertainty in image motion processing was consistently larger than uncertainty in depth processing for all observers. This was confirmed by testing the difference between the two noise estimates of the BVN2 model from each observer (Figure 7) using bootstrapped data from the matching task (mean difference of 5.5% with 95% confidence interval CI 95% = [3.3, 7.6], *t*(4) = 7.16., *p* = 0.002). The bootstrapped data clearly suggest increased noise for monocular motion compared to dynamic depth processing in all participants. Despite inter-individual variability [54,55,56] the results indicate contributions from two separate (BVN2) rather than a single source of noise (BVN1).

In Exp. 1 we varied orientation disparity of the line stimulus around the vertical to investigate the effect of slant of the line stimulus on perceived 3D motion direction. This resulted in a range of perceived 3D motion directions that were centred on two horizontal trajectories with elevation of 0 deg (±IOVD). In Exp. 2 we used oblique line stimuli and varied orientation disparity in the tilted line stimuli. We reasoned that in Exp. 1 vertical line stimuli with horizontal motion direction may have served as a reference for the other motion trajectories. In Exp. 2 we increased the possible range of motion directions and used tilted and slanted oblique stimuli. Removing a vertical line stimulus with horizontal motion direction may have increased ambiguity in our observers.

### 3.2. Results of Experiment 2

We investigated perceived motion directions of tilted and slanted line stimuli. The moving line stimuli were tilted by either 45 or 135 deg from vertical and systematically slanted in depth about their oblique tilt axes. Interocular velocity difference (IOVD) had the same value as in Exp. 1 but was defined along the oblique tilt axes rather than the horizontal *x*-axis. Sign of IOVDs (±0.46 deg/s), tilt (45 and 135 deg), and orientation disparities (±6 deg in steps of 2 deg) gave a total of 2 × 2 × 7 = 28 possible 3D trajectories, twice as many as in Exp. 1. Possible 3D trajectories were centred on four different oblique trajectories, suggesting 3D line motion in each octant of a 3D co-ordinate system (e.g., near bottom-left to far top-right) with the start point as the origin.

Conditions in which the line orientation and motion trajectories were mirror-symmetric (45 deg and 135 deg line stimulus with opposite sign of the IOVD) gave similar adjustments of perceived 3D motion direction. The adjustments of mirror-symmetric trajectories and line orientations were collapsed to increase the number of observations per condition. Positive longitude angles, measured on the fronto-parallel *x*-*y* plane, described motion trajectories towards the observer (+IOVD/near) and negative longitude angles indicate trajectories away from the observer (−IOVD/far). Positive latitude angles describe trajectories pointing up, relative to the horizontal observer’s line of sight, whereas negative latitude angles describe trajectories pointing down.

**Figure 8:**
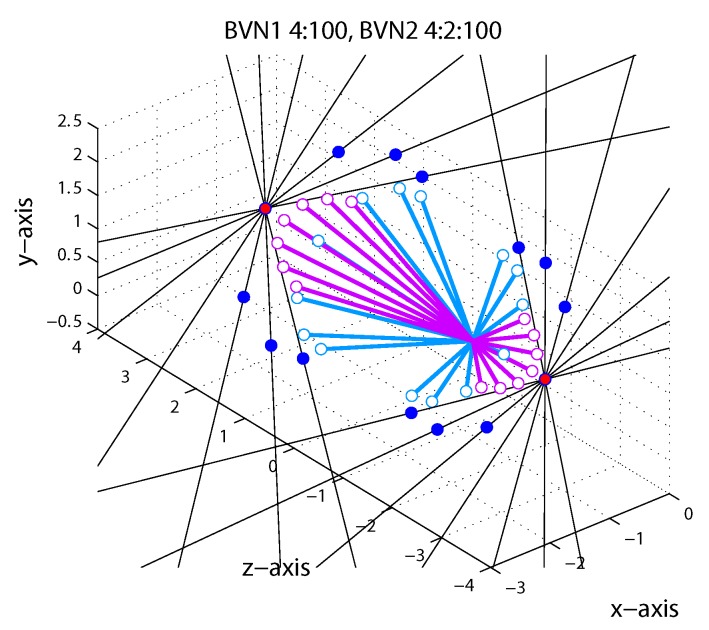
Model predictions for line stimuli in Exp. 2. Each of the seven black hairlines corresponds to a constraint (ICP) for an upwards trajectory to the left and away from the observer (−IOVD/far) and to the left and towards the observer (+IOVD/near). Red circles correspond to the cyclopean average (CA) and dark blue circles to vector normal (VN) predictions. Light blue and light purple open circles correspond to BVN1 and BVN2 predictions, respectively. See text for details.

As in Exp. 1 the binocular velocity constraints (ICPs) with different orientation disparity and the corresponding model predictions are illustrated in Figure 8. Predictions of the CA strategy (red circles) remained constant for oblique line stimuli with different orientation disparities. Predictions of the VN strategy gave endpoints who fan out in 3D (dark blue circles). Predictions of the Bayesian vector normal models (BVN) are shown as open circles with lines attached to illustrate the predicted 3D trajectories emanating from the start point at (0,0,0). The BVN1 model with noise ratio σ:$\sigma_{0}$ = 4:100 (light blue) makes motion predictions that diverge less than the VN strategy whereas BVN2 model with noise ratio $\sigma_{m}$:$\sigma_{d}$:$\sigma_{0}$ = 4:2:100 (light purple circles) describes endpoints that are clustered around the CA prediction (red circle). With increasing noise levels the BVN1 and BVN2 model predict shorter trajectories (speeds) at reduced longitudinal angles. With very small and unbalanced noise $\sigma_{m}$ > $\sigma_{d}$ BVN2 trajectories approximate the constant prediction of the CA strategy. (The reader can explore predictions for different noise settings using the MatLab code provided under osf.io/2j6sq).

**Figure 9:**
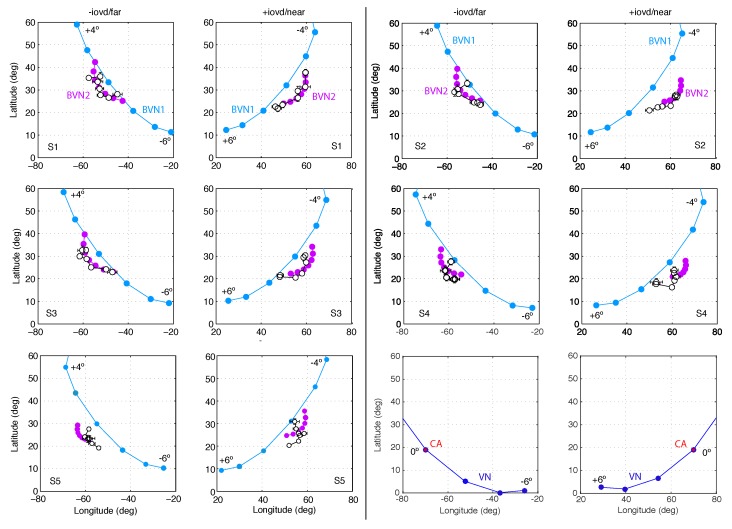
Motion directions in Exp. 2. In two columns pairs of graphs are shown for Observer S1 to S5 with −IOVD/far on the left +IOVD/near on the right. In each graph latitude is plotted against longitude angles. Circles connected by a line indicate motion directions for the seven line stimuli slanted in depth from oblique with orientation difference $-6^{{}^{\circ}}$ to $+6^{{}^{\circ}}$ in steps of $2^{{}^{\circ}}$. Black open circles represent observed longitude and latitude angles, averaged across repeated trials. Horizontal and vertical error bars denote ±1 SEM. Light blue and light purple circles denote angular estimates of the best-fitting BVN1 and BVN2 model, respectively. The last pair of graphs illustrates the geometric predictions of VN (blue circles) and the CA (red circles).

As in Exp. 1, tilted line stimuli slanted in depth about the oblique tilt axis resulted in perceived 3D motion directions that had similar characteristics across observers. Observer S1 had 6.4% noise for BVN1 compared to 12.7% motion noise and 5.3% dynamic depth noise for BVN2 (see Figure 9 and Table A1). The likelihood ratio was statistically significant ($\chi^{2}\left(1\right)$ = 6.65, *p* = 0.01) and the Bayes factor (BF = 7.4) indicated strong evidence in favour of BVN2. Observer S2 had 6.2% noise according to the BVN1 model and 13.8% monocular motion noise and 5.0% dynamic depth noise according to the BVN2 model. The likelihood ratio was also statistically significant ($\chi^{2}\left(1\right)$ = 5.8, *p* = 0.016) with a Bayes factor (BF = 4.9) in favour of BVN2. Similar effects emerged for Observer S3 ($\chi^{2}\left(1\right)$ = 6.6, *p* = 0.01; BF = 7.3), Observer S4 ($\chi^{2}\left(1\right)$ = 6.1, *p* = 0.014; BF = 5.5) and Observer S5 ($\chi^{2}\left(1\right)$ = 6.9, *p* = 0.009; BF = 8.5).

The results of Exp. 2 confirm that geometric defaults, such as the parameter-free vector normal (VN) and cyclopean average (CA), are too rigid to explain perceived 3D motion directions under ambiguity. The BVN2 model captured key characteristics of the data but individual data fits were slightly worse than in Exp. 1, showing systematic misalignments between model predictions and observed data. This may be due to the lack of stimuli with cardinal alignment (vertical, horizontal) and the increased number of stimulus conditions in Exp. 2. Nevertheless, BVN2 provided a significantly better model fit than BVN1 for perceived 3D motion directions of slanted lines moving on oblique axes.

**Figure 10:**
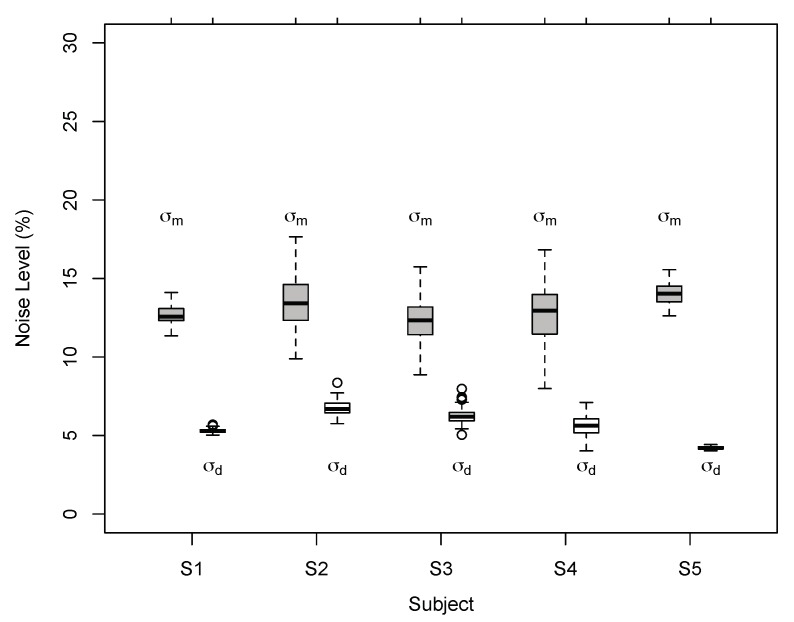
Individual noise estimates for BVN2 in Exp. 2. Estimates of monocular motion ($\sigma_{m}$) and dynamic depth ($\sigma_{d}$) for Observer S1 to S5 for bootstrapped data. Thick horizontal lines denote medians, boxes the second and third quartile with error bars (whiskers) extending 1.5 times of the interquartile range. Open circles denote outliers. All pairwise differences between individual motion and depth noise estimates were statistically significant (*p* < 0.0002, two-tailed *t*-test, Bonferroni-adjusted).

As in Exp. 1 the results suggest that perceived 3D line motion directions deviated systematically from VN and CA. Perceived motion directions varied between observers but showed characteristics that are more compatible with the BVN2 model. Model comparisons favoured BVN2 over BVN1 in all observers of Exp. 2. As in Exp. 1, noise estimates from bootstrapped data (Figure 10) were significantly larger for monocular motion than dynamic depth processing across all five observers (mean noise difference 7.4%, CI 95% = [5.7, 9.2]; t(4) = 11.8, *p* = 0.0003). The estimates of the noise ratios $\sigma_{m}$:$\sigma_{0}$ and $\sigma_{d}$:$\sigma_{0}$ of the best-fitting BVN2 model suggest that for tilted and slanted line stimuli noise in image motion processing was larger compared to noise from dynamic depth processing across observers.

The slightly increased noise ratios for motion and reduced noise ratio for dynamic depth processing of the BVN2 model may reflect characteristics of oriented line stimuli translating on oblique trajectories in depth. Although interocular velocity difference was the same as in Exp. 1, IOVD in Exp. 2 was defined for oblique trajectories, resulting in perceived motion directions centred on four oblique rather than two horizontal 3D trajectories. Similarly, orientation disparity, measured in angular degrees between line images, assumed the same values as in Exp. 1 but were defined around oblique axes rather than the horizontal axis. Compared to the line stimuli in Exp. 1 the oblique line stimuli had the same oriented disparities but larger horizontal and/or vertical disparities. Increasing the dynamic range of horizontal disparities in the line stimuli of Exp. 2 resulted in BVN predictions that diverged more than in Exp. 1.

BVN2 noise estimates for motion processing $\sigma_{m}$ were similar in both experiments. The noise estimates for dynamic depth processing $\sigma_{d}$ were slightly reduced in Exp. 2 and consistently smaller than $\sigma_{m}$. It seems likely that orientation disparities for lines slanted in depth about the obliques increased the range of binocular disparities in the stimuli [57,58,59]. We speculate that the absence of line stimuli with horizontal motion direction and the increase in slant may have reduced uncertainty in depth processing.

## 4. Discussion

The results of the two experiments suggest that perceived 3D line motion directions deviate systematically from deterministic or geometric default strategies such as the vector normal (VN) and cyclopean average (CA) [19]. The empirical results are better described by a geometric-statistical model of 3D motion perception. In particular the BVN2 model is more flexible and is therefore better suited to capture perceived 3D motion directions.

Interestingly, dynamic depth processing affected 3D motion perception more strongly than monocular motion processing. This is difficult to reconcile with existing 3D motion models that employ monocular motion encoding or early stereo-motion encoding as inputs. These models assume that encoding is based on motion energy and joint motion and disparity energy which suggests a single source of noise for motion and disparity processing [31,42,43,44,45]. According to these computational models and in contrast to our results, motion should be the dominant input rendering noise estimates that are smaller, or at least equal to, the noise estimates of dynamic depth processing.

We suggest that in the present experiments monocular motion processing of line stimuli carried substantial ambiguity and uncertainty. Unlike random-dot motion stimuli that typically provide multiple unambiguous motion cues, a single line stimulus behind an aperture introduces ambiguous local motion. We speculate that dynamic binocular disparity between the moving line stimulus and the static aperture may have contributed more strongly to perceived 3D motion direction than the image gradients. It seems likely that “tracking of binocular features” with horizontal and vertical disparity, such as T-junctions between the line stimulus and the aperture [60,61] helped to disambiguate 3D line motion direction. This would promote dynamic disparity over monocular motion input and is compatible with the BVN2 model that combines motion and dynamic disparity input weighted by its uncertainty.

### Limitations and Implications

The motion-direction matching task was difficult to perform. The task required extensive training and one observer had to be excluded. The small sample size and number of trials made the data unsuitable for a hierarchical estimation of noise parameters of likelihoods and prior [29,32]. Nevertheless, our results may be regarded as a proof-of-concept awaiting further empirical validation.

The Bayesian vector normal model assumes a spherical Gaussian distribution as 3D motion prior. This conjugate prior was selected for computational convenience, since estimating velocity has a closed analytic form. It is possible that other weak motion priors with heavier tails, i.e., the lognormal, Laplace, or Cauchy distribution, may be more appropriate as suggested by studies on 2D velocity discrimination [29,62]. However, the specific form of a weak motion prior should have little effect on the results as long as the prior is isotropic.

The BVN models are based on horizontal disparity as input. However, it seems likely that the visual system encodes oriented rather than horizontal disparity from local receptive fields [58,59,63]. Using receptive fields with (dynamic) oriented disparity as input may improve model predictions.

The results of the present study suggest that the visual system needs to combine monocular motion with dynamic binocular disparity when estimating 3D velocity. A Bayesian model with a spherical motion prior and independent noise in motion and depth processing suggests that motion and depth constraints are integrated at a relatively late processing stage. Indeed Bayes-optimal cue integration [64] follows the principle of late commitment [2] and provides a flexible scheme for integrating constraints from early motion and disparity encoding in V1, additional constraints from intermediate processing in V2 to V4 [65,66,67], and possibly ecological constraints from higher processing stages. The separate pathways that connect V1 directly to human MT+/V5 and indirectly via V2 and V3 [68,69] are good candidates for separate and independent processing streams [70]. This anatomical and functional divide suggests relatively late integration of motion and dynamic disparity information [71,72,73,74,75,76] The advantage of this architecture is that weighted contributions from motion and (dynamic) depth processing, together with motion prediction and other top-down processing, can help to disambiguate local motion constraints and solve the 3D aperture problem.

## 5. Conclusions

What enables the visual system to perceive 3D motion and to infer arbitrary directions and speeds of a moving object? It has been suggested that the visual system exploits many cues in concert to make this difficult inference as reliable and veridical as possible [77]. Local velocity estimates need to be accurate yet flexible in order to accommodate a wide range of stimulus constraints [2]. The diverse set of effective local and global cues [78] and spatial-temporal tuning characteristics of 3D motion perception [79,80,81] suggest the involvement of relatively late processing stages in a hierarchical visual system [39,82]. In contrast, many physiological and computational models of 3D motion assume motion input or early integration of motion and disparity input as the basis of binocular 3D motion perception.

The probabilistic approach pursued here provides a flexible framework for the integration of local motion and dynamic depth information. We suggest that the aperture problem of 3D motion is solved late in the visual hierarchy by using minimal distance in 3D as a general principle when disambiguating local velocity constraints. Using this principle, the Bayesian vector normal model with motion and dynamic disparity as input (BVN2) gave a comprehensive account of individual motion percepts. The two model parameters revealed that dynamic binocular depth processing contributed more strongly to perceived 3D line motion direction than monocular motion processing. Future research needs to demonstrate whether observers do indeed integrate monocular motion, dynamic binocular disparity, as well as other cues in a Bayes-optimal fashion. The present model provides a conceptual framework for late integration of motion and dynamic depth processing but requires further empirical and neuroscientific validation.

## Appendix A

### Appendix A.1. Viewing Geometry

Any point p=(x,y,z) in Cartesian co-ordinates with origin O=(0,0,0) can be transformed into (ordinary) spherical co-ordinates (α,β,∥v∥) using q=(x,0,z) and r=(x,0,0) as base vectors in order to determine longitude angle α on the horizontal plane through the eyes

(A1) $$\begin{array}{cc} \alpha = & \arccos\left(\frac{q\cdot r}{∥q∥\;∥r∥}\right) \end{array}$$

where · is the scalar or dot product between vectors and ∥…∥ the norm of a vector. Similarly, for base vectors v=(x,y,z) and q=(x,0,z) latitude angle β is given by

(A2) $$\begin{array}{cc} \beta = & \arccos\left(\frac{s\cdot q}{∥v∥\;∥q∥}\right) \end{array}$$

For the sake of simplicity we assume a fixed viewing geometry where the eyes are in primary position and the fixation point straight ahead of the observer. The nodal points of the eyes and the fixation point are known and define a horizontal plane. In the following, image projections onto an image plane, nodal points of the eyes and a fixation point are known to the observer [57]. Line motion direction is computed as a vector in Cartesian co-ordinates but perceived directions and model predictions are reported in (ordinary) spherical co-ordinates in terms of longitude α and latitude β.

A point behind the fixation cross at the centre of the aperture serves as the origin O=(0,0,0) of the Cartesian coordinate system. As usual the *x* and *y*-axis describe the horizontal and vertical, respectively. Depth along the line of sight is represented by the *z*-axis.

Geometrically the left and right eye (velocity) constraint planes are defined by the nodal point of the left and right eye and corresponding 2D motion constraints on the retina. As a matter of convenience we use a single fronto-parallel screen at viewing distance *D* as the image plane rather than projections onto the retinae on the back of the left and right eye.

### Appendix A.2. Velocity Constraints

If the local aperture is small in relation to the viewing distance and if the line moves in the vicinity of the fixation point on the median plane then inverse projection of the left and right eye velocity constraints can be approximated by linear hyper-planes cutting through 3D space. Each velocity constraint plane is defined by the nodal point in each eye and the corresponding 2D motion constraint line on the retina or equivalently on the image plane in front of the eyes. Two points $p_{1}$ and $p_{2}$ on this image plane define a line (2D velocity constraint) projected into the left eye

(A3) $$\begin{array}{cc} p_{1}= & \left(t_{L},0,0\right),\\ p_{2}= & \left(t_{L}-\cos\left(\theta_{L}\right),\sin\left(\theta_{L}\right),0\right) \end{array}$$

where $t_{L}$ denotes the horizontal component of the 2D motion gradient in the image plane and line orientation $\theta_{L}$ describes the angle between horizontal *x*-axis and line image. Similarly, two points $p_{3}$ and $p_{4}$ on the image plane define a line (2D velocity constraint) projected into the right eye

(A4) $$\begin{array}{cc} p_{3}= & \left(t_{R},0,0\right),\\ p_{4}= & \left(t_{R}-\cos\left(\theta_{R}\right),\sin\left(\theta_{R}\right),0\right) \end{array}$$

where $t_{R}$ denotes the horizontal translation of the image-motion gradient and line orientation $\theta_{R}$ describes the angle between horizontal *x*-axis and line image.

Next, we define interocular velocity difference (IOVD) of the line stimulus as the difference between translations along the horizontal (oblique in Exp. 2) axis $\left(t_{L}-t_{R}\right)$, and orientation disparity as the difference between orientations $\left(\theta_{L}-\theta_{R}\right)$ between the line images on the fronto-parallel image plane at viewing distance *D*.

The nodal point of the left and right eye remains constant and is defined as

(A5) $$\begin{array}{cc} p_{L}= & \left(-i/2,0,-D\right),\\ p_{R}= & \left(+i/2,0,-D\right) \end{array}$$

where *i* = 6.5 cm denotes interocular distance and viewing distance *D* = 55 cm describes the distance between cyclopean point *C* between the two eyes and the fixation point *F* on the image plane (see Figure 1).

Then the (Hessian) normal of the left eye and right eye constraint plane can be expressed as

(A6) $$\begin{array}{cc} n_{L}= & \frac{\left(p_{2}-p_{L}\right)\times \left(p_{1}-p_{L}\right)}{∥\left(p_{2}-p_{L}\right)\times \left(p_{1}-p_{L}\right)∥}\\ n_{R}= & \frac{\left(p_{4}-p_{R}\right)\times \left(p_{3}-p_{R}\right)}{∥\left(p_{4}-p_{R}\right)\times \left(p_{3}-p_{R}\right)∥} \end{array}$$

where × denotes the cross product and ∥…∥ the norm.

Two planes always intersect in a line as long as they are not parallel [20]. To specify the intersection of constraint planes (ICP), it is necessary to identify a particular point on the constraint line. This is equivalent to determining a point v that belongs to both constraint planes and satisfying the equations

(A7) $$\begin{array}{cc} n_{L}\cdot v+d_{L}= & 0\\ n_{R}\cdot v+d_{R}= & 0 \end{array}$$

where · denotes the dot product and $d_{L}$ and $d_{R}$ are the distances from the constraint plane to the origin, respectively. In general this linear system is under-determined because there are only two equations and v has three unknowns. We introduce further constraints in the next section.

### Appendix A.3. Brightness and Velocity Constraints

In analogy to local 2D motion gradients in an image 3D motion gradients can be approximated by first-order Taylor series expansion using brightness constraints.

(A8) $$\begin{array}{cc} v^{T}\nabla L+L_{t}= & 0,\\ v^{T}\nabla R+R_{t}= & 0 \end{array}$$

where $v=(v_{x},v_{y},v_{z})$ denotes the (unknown) 3D velocity of the line stimulus that complies with both velocity constraint planes. The spatial and temporal gradient ∇L and $L_{t}$ and ∇R and $R_{t}$ describe the constraint plane for the left and right eye, respectively.

The (Hessian) normals of these constraint planes can be expressed by their respective spatial and temporal gradients

(A9) $$\begin{array}{c} n_{L}=\frac{\nabla L}{∥\nabla L∥}\frac{L_{t}}{∥\nabla L∥},\\ n_{R}=\frac{\nabla R}{∥\nabla R∥}\frac{R_{t}}{∥\nabla R∥} \end{array}$$

Since intensity gradients are measured on an *x*-*y* image or surface, the spatial and temporal gradients in depth along the *z*-axis are undefined. However, we canonically extend the definition of 2D line gradients to 3D planes by assuming constant gradients in the co-ordinates (x,y,z) that project onto the same image velocity constraint–effectively defining a brightness constraint in 3D by inverse projection through the nodal point of the left and right eye. Inverse projection defines two velocity constraint planes cutting through 3D space. Each plane describes all possible oriented end positions and therefore velocities of a line moving in 3D given the observed image motion in the corresponding eye. The intersection of the left and right eye constraint planes (ICP) defines a velocity constraint line in 3D. Monitoring ICPs in 3D space over time mimics a stereo-first motion system that encodes dynamic binocular disparity constraints. This implies that a visual system that relies on ICPs may not only monitor independent monocular motion but also binocular disparities over time when estimating 3D velocity.

### Appendix A.4. Bayesian Vector Normal Model

The velocity constraint planes and their intersection may be noisy due to motion encoding, disparity processing, refraction error, micro-saccades and other sources of noise. We further assume that for relatively slow motion the temporal derivatives $L_{t}$ and $R_{t}$ have negligible noise whereas the spatial derivatives ∇L and ∇R contain additive i.i.d. Gaussian noise η

(A10) $$\begin{array}{cc} \nabla \tilde{L}= & \nabla L(x,y,z)+\eta (x,y,z),\\ \nabla \tilde{R}= & \nabla R(x,y,z)+\eta (x,y,x) \end{array}$$

Given that for small displacements in 3D the intensity of a line or edge does not change (establishing a motion constraint equation in 3D) the following expressions for the likelihoods in the left and right eye are also normally distributed

(A11) $$\begin{array}{cc} v^{T}\nabla \tilde{L}+L_{t}∼ & N(0,\Sigma ),\\ v^{T}\nabla \tilde{R}+R_{t}∼ & N(0,\Sigma ) \end{array}$$

where N(0,Σ) denotes a multivariate Gaussian distribution centred on 0 with diagonal covariance matrix $\Sigma =\left[\begin{array}{ccc} \sigma_{x}^{2} & 0 & 0\\ 0 & \sigma_{y}^{2} & 0\\ 0 & 0 & \sigma_{z}^{2} \end{array}\right]$.

If the interocular distance is sufficiently small compared to viewing distance then adding noise to the spatial gradients reflects uncertainty in 3D line motion processing. The added Gaussian noise blurs the left and right eye velocity constraint plane and their intersection (ICP) gives rise to a volumetric distribution around the ICP.

If 3D velocity v of the moving line is known then the likelihood of observing a particular velocity constraint for the left and right eye can be expressed as

(A12) $$\begin{array}{c} p\left(\nabla \tilde{L},L_{t}|v\right)=\frac{1}{\sqrt{{\left(2\pi \right)}^{3}\det\left(\Sigma \right)}}\exp\left(-\frac{1}{2}{\left(v^{T}\nabla L+L_{t}\right)}^{T}\Sigma^{-1}\left(v^{T}\nabla L+L_{t}\right)\right),\\ p\left(\nabla \tilde{R},R_{t}|v\right)=\frac{1}{\sqrt{{\left(2\pi \right)}^{3}\det\left(\Sigma \right)}}\exp\left(-\frac{1}{2}{\left(v^{T}\nabla R+R_{t}\right)}^{T}\Sigma^{-1}\left(v^{T}\nabla R+R_{t}\right)\right) \end{array}$$

Assuming that most features and objects in a scene are perceived as stationary or as moving slowly on a 3D trajectory independent of the observer then a spherical Gaussian provides a plausible world prior for 3D motion perception [27].

(A13) $$\begin{array}{cc} p(v)= & \frac{1}{\sqrt{{\left(2\pi \right)}^{3}\sigma_{0}^{3}}}\exp\left(\frac{{-v}^{T}v}{2\sigma_{0}^{2}}\right) \end{array}$$

We derive the posterior distribution by combining likelihood constraints and prior according to Bayes rule. We can drop the denominator because this expression is independent of velocity v. We can drop the denominator because this expression is independent of velocity v. We then approximate the posterior by the product of the likelihoods and the prior:

(A14) $$\begin{array}{cc} p(v|\left(\nabla \tilde{L},L_{t}\right),\left(\nabla \tilde{R},R_{t}\right))\propto & p\left(\nabla \tilde{L},L_{t}|v\right)p\left(\nabla \tilde{R},R_{t}|v\right)\;p\left(v\right) \end{array}$$

In order to find the mode or maximum a posteriori (MAP) estimate of 3D velocity, we take the negative logarithm of the posterior, differentiate it with respect to vector v and set the derivative equal to zero. The logarithm of the posterior is quadratic in v so that the solution can be written in closed form using standard linear algebra.

(A15) $$\begin{array}{c} \frac{d}{dv}\left(-\log\left[p\left(v|\left(\nabla \tilde{L},L_{t}\right),\left(\nabla \tilde{R},R_{t}\right)\right)\right]\right)=0 \end{array}$$

Solving for 3D velocity v gives

(A16) $$\begin{array}{cc} \hat{v}= & -{\left(M+\frac{1}{\sigma_{0}^{2}}I\right)}^{-1}b \end{array}$$

where I is the 3×3 identity matrix and

(A17) $$\begin{array}{cc} M= & \left(\nabla L\Sigma^{-1}\nabla L^{T}+\nabla R\Sigma^{-1}\nabla R^{T}\right),\\ b= & \left(\nabla L\Sigma^{-1}L_{t}+\nabla R\Sigma^{-1}R_{t}\right) \end{array}$$

and $\Sigma^{-1}$ the inverse of the diagonal covariance matrix. If matrix M is nonsingular we obtain an analytical solution with a unique 3D velocity estimate $\hat{v}=\left({\hat{v}}_{x},{\hat{v}}_{y},{\hat{v}}_{z}\right)$ for ambiguous line motion behind a local aperture.

The Bayesian vector normal model with a single noise parameter (BVN1) and noise ratio σ:$\sigma_{0}$ describes uncertainty between the velocity likelihood and a constant prior across all three components. Alternatively, if the Bayesian vector normal model has two independent but not necessarily identical noise parameters (BVN2) then $\sigma_{m}$ captures uncertainty of monocular motion processing in the (retinal) *x*-*y* image plane independent of depth processing $\sigma_{d}$ along the *z*-component. Introducing two noise ratios $\sigma_{m}$:$\sigma_{0}$ and $\sigma_{d}$:$\sigma_{0}$ in the BVN2 model implies that monocular motion and binocular depth processing are based on different processing streams with independent but not necessarily identical noise sources. A third version of the Bayesian vector normal model with three independent noise parameters (BVN3) was not considered here but may be of interest for object motion with distinguishable noise sources in all three components.

### Appendix A.5. Model Fit and Model Comparison

The Bayesian vector normal model with one (BVN1) or two parameters (BVN2) computes perceived 3D motion direction as a Bayesian inference. The BVN1 model with small noise parameter σ approximates the geometric predictions of the vector normal (VN), whereas the BVN2 model with two parameters can accommodate the vector normal (VN), cyclopean average (CA), as well as intermediate predictions. The two parameters of the BVN2 model quantify how strongly perceived motion direction is influenced by noise in monocular motion and dynamic depth processing. Fitting the BVN2 model with two noise parameters to a single perceived motion direction in terms of longitude and latitude angle would be trivial. However, fitting the BVN2 model to 2 × 7= 14 different line motion directions with 28 observed angles in Experiment 1 and to 2 × 2 × 7 = 28 different line motion directions with 56 observed angles in Experiment 2 establishes a comprehensive model test.

By fitting the independent noise parameter $\sigma_{m}$ for monocular motion and $\sigma_{d}$ for dynamic depth processing in the BVN2 model while keeping noise in the prior $\sigma_{0}$ = 100% constant, we can estimate the noise ratio between likelihood and prior in motion (*x*-*y*-axes) and depth (*z*-axis) for individual observers and different experiments (vertical and oblique). Maximum-likelihood estimates and $\chi^{2}$-fits were obtained for the BVN1 and BVN2 model assuming normal and independent error distributions for longitude and latitude and using the fminsearch() routine in MATLAB (MathWorks, Natick, MA, USA) to minimise the squared differences between both observed and predicted angles. We compared the BVN1 model with spherical noise against the BVN2 model with diagonal noise by performing a conventional likelihood-ratio test for nested models (LR-statistic, approximately $\chi^{2}$-distributed with (2 − 1) = 1 degree of freedom). We approximated the Bayes factor (BF) using Bayesian information content (BIC). The BIC for model $M_{i}$ is defined as

(A18) $$\begin{array}{cc} BIC(M_{i})= & -2\log\left(L_{i}\right)+k_{i}\log\left(n\right) \end{array}$$

where $L_{i}$ and $k_{i}$ is the maximum likelihood and number of free parameters of model $M_{i}$, respectively, and *n* is the number of observations [83,84] Assuming that the prior odds for model $M_{1}$ (BVN1) and $M_{2}$ (BVN2) are the same the Bayes factor is the ratio of the prior predictive probabilities. Prior predictive probabilities, the probability of data *D* given a model $M_{i}$, can be approximated by $Pr\left(D|M_{i}\right)\approx \exp\left(-BIC\left(M_{i}\right)/2\right)$. Comparing two competing models using BIC approximations gives

(A19) $$\begin{array}{c} BF_{12}=\frac{Pr(D|M_{1})}{Pr(D|M_{2})}\approx \exp\left(\Delta BIC_{21}/2\right) \end{array}$$

where $\Delta BIC_{21}$ = $BIC\left(M_{2}\right)-BIC\left(M_{1}\right)$. The approximate Bayes factor $BF_{12}$ indicates the odds in favour of the BVN1 model compared to the BVN2 model. In Table A1 we report $BF_{21}=BF$ in favour of the BVN2 model.

**Table A1:** Noise ratios (%), goodness-of-fit ($\chi^{2}$), and model comparison for Bayesian vector normal model with one (BVN1) and two noise parameters (BVN2) in Exp. 1 (slanted lines) and Exp. 2 (tilted and slanted lines). Model comparison in terms of likelihood ratio LR and Bayes factor BF.

Obs.	BVN1		BVN2			Mod. Comp.	
Exp. 1	σ:$\sigma_{0}$	$\chi_{12}^{2}$	$\sigma_{m}$:$\sigma_{0}$	$\sigma_{d}$:$\sigma_{0}$	$\chi_{11}^{2}$	LR	BF
S1	7.03	93.0	8.97	6.85	21.7	1.89	0.69
S2	6.42	227.4	14.48	6.03	4.32	8.27	16.67
S3	6.68	182.2	13.53	6.35	12.0	5.08	3.39
S4	6.20	54.7	9.55	6.00	7.01	3.28	1.38
S5	6.19	24.96	10.22	5.95	7.63	3.37	1.44
Exp. 2	σ:$\sigma_{0}$	$\chi_{24}^{2}$	$\sigma_{m}$:$\sigma_{0}$	$\sigma_{d}$:$\sigma_{0}$	$\chi_{23}^{2}$	LR	BF
S1	6.41	486.3	12.71	5.31	15.2	6.65	7.43
S2	6.18	570.8	13.77	5.00	29.3	5.81	4.89
S3	5.58	859.1	11.20	4.48	24.9.0	6.62	7.33
S4	4.67	632.9	11.01	3.36	27.8	6.06	5.54
S5	5.59	1741	13.99	4.21	46.6	6.92	8.52
